# Imaging Scatterometry with Extrapolation of Missing BRDF Data for Materials Used in Laser Material Processing

**DOI:** 10.3390/s21010008

**Published:** 2020-12-22

**Authors:** Adrian Zakrzewski, Piotr Jurewicz, Michał Ćwikła, Piotr Koruba, Jacek Reiner

**Affiliations:** Faculty of Mechanical Engineering, Wrocław University of Science and Technology, Wybrzeże Stanisława Wyspiańskiego 27, 50-370 Wrocław, Poland; piotr.jurewicz@pwr.edu.pl (P.J.); michal.cwikla@pwr.edu.pl (M.Ć.); piotr.koruba@pwr.edu.pl (P.K.); jacek.reiner@pwr.edu.pl (J.R.)

**Keywords:** imaging scatterometry, BRDF reconstruction, reflection distribution

## Abstract

Imaging scatterometry is a method for determining the reflection distribution based on bidirectional reflectance distribution function (BRDF) measurements. However, it has a well-known limitation that results obtained by imaging scatterometry for small illumination angles are practically useless. Therefore, we propose an approach for reconstruction of the reflection distribution based on a series of measurements at different illumination angles and extrapolation of the missing results to overcome this limitation. The developed algorithm was validated using bidirectional transmittance distribution function (BTDF) measurements. The BRDF measurements were carried out for materials that are commonly used in laser material processing, i.e. substrates and functional coatings. The obtained data were subsequently used to determine the total reflection intensity from all considered materials, which were characterized by reconstructed distributions.

## 1. Introduction

Analysis of materials surfaces becomes crucial because of increasing quality requirements and repeatability control of components production, especially in the case of structured materials [1,2] or performance quality of typical optical components [3,4]. The surface quality is not only related to roughness but also to local defects and every type of bulk inhomogeneity. These aspects are directly associated with the kind of light scattering on the material’s surface. In case of smooth surfaces, analysis based on specular reflection is sufficient in order to assess the surface quality. However, in the case of most studies on surface quality, analysis of diffuse reflection is required. Overall, the reflection distribution provides comprehensive information about the directivity and intensity of the light reflected from the sample.

Generally, the intensity of the light reflected from metal surfaces, which is commonly used in laser processes, is available as a function of wavelength and illumination angle (α) [5]. However, these results apply only to specular reflection [6], which is rarely a dominant property of the engineering materials. In most cases, the specular reflection is combined with diffuse reflection. Moreover, such data are unavailable for functional coatings deposited on the metal substrates, especially if different production process parameters are taken into account.

The optical approach designed to determine the reflection distribution as a function of α are scatterometry methods. These group of methods are non-contact and non-destructive, and in addition allow a higher rate of data acquisition and the possibility of measuring samples with much larger dimensions [7]. The result of scatterometry measurements is a bidirectional function describing spatial distribution of light scattering—the bidirectional scattering distribution function (BSDF) [8]. This function can describe the reflection, as well as the transmission distribution. In a particular case it is called the bidirectional reflectance/transmittance distribution function (BRDF/BTDF) [9].

There are several methods of scatterometry that differ in their complexity. The most popular methods use a gloss meter [10], a multi-angle colorimeter [11] and a gonioreflectometer [12], which allow obtaining comprehensive information of light distribution scattering of the surface. However, these methods are time-consuming, which is their main drawback. A faster alternative is the combination of an integrating hemisphere and a CCD camera called imaging scatterometery (IS) [13]. This combination resolves the issue of a single, mobile detector in the gonioreflectometer, since in the case of IS the information is obtained simultaneously for each of the millions of camera pixels. As a first, the IS method was applied for determining the BSDF in the Parousiameter device patented by Philips [14]. The implementation of this patent was carried out by Radiant Imaging (Radiant Zemax from 2012); they constructed the commercial scatterometer IS-SA (Imaging Sphere for Scatter and Appearance measurements) device [15].

In this paper, we present an approach for determination of the reflection distribution based on IS measurements. However, due to a well-known limitation of imaging scatterometry, the BRDF data obtained for small α related to the surface are practically useless [16,17]. Therefore, we present a new approach for reconstruction of missing BRDF data. The proposed algorithm is based on fitting a modified Lorentzian function to the correct measurements (carried out for larger α) and extrapolating the parameters of this function for corrupted measurements. The algorithm has been validated based on the BTDF measurements results. The BRDF measurements were carried out for four metallic materials, which are commonly used in laser material processing, in various surface conditions, i.e. substrates and clads. Finally, the total reflection intensity from the materials characterized by reconstructed distribution was determined.

## 2. Materials and Methods

### 2.1. Samples Preparation

Four metallic materials in various surface conditions were selected for scatterometry measurements. A mirror (substrate made of N-BK7 material with a silver coating) was used as a reference. It was selected because of its well-defined and almost constant reflectance value of over 97.5% for 575 nm [18]. Materials that were compared included a substrate made of mild steel S420MC and a laser with a deposited coating made of stainless steel 316L (S420MC_316L) [19]. The considered issue relates to a common case when steel characterized by sensitivity to corrosion and atmospheric conditions is cladded with a layer of stainless steel that protects the exposed surface exposed from degradation. For the S420MC steel substrate, two methods of surface preparation were used: surface degreasing without mechanical removal of the oxide layer appearing during metallurgical production (S420MC_oxidized) and mechanical removal of the oxide coating with abrasive paper (S420MC).

The surface roughness of the materials was determined according to ISO 25,178 [20] using an Olympus LEXT 3D Measuring Laser Microscope OLS4000 with 50× magnification. Table 1 shows the basic area roughness parameters for each of the examined surfaces.

### 2.2. IS Method

The IS measurements of samples were carried out using an IS-SA equipped with a 16-bit imaging camera with a resolution of 1024 × 1024 px. The radius of the integration sphere was 30 cm. The device accuracy in the range of BRDF values was 5% [16]. A DC950 illuminator with a fiber optic adapter was used as a light source. The total radiation power was 150 W, with the local maximum intensity occurring in the visible range. Additionally, a 575 nm narrow bandpass filter was used. Prior to the main measurements, the device was calibrated using a 6 mm diameter clear, Lambertian pattern aperture. The scatterometry measurements were performed three times and averaged in the range of α from 0° to 60° with a 5° step.

**Figure 1:**
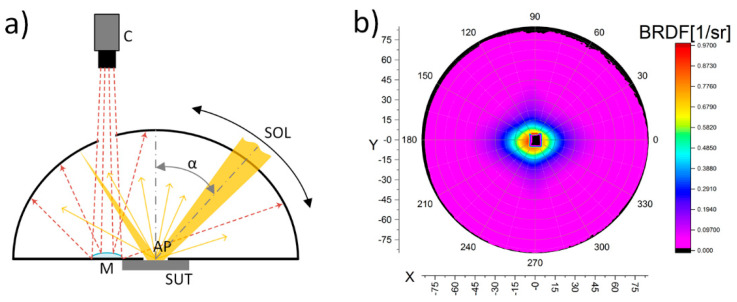
Visualization of negative obscuring effect: (**a**) the Imaging Sphere for Scatter and Appearance (IS-SA) principle of operation and (**b**) the bidirectional reflectance distribution function (BRDF) plot for α = 0°.

Despite many advantages (described in Section 1), IS-SA also has significant limitations such as the effect of obscuring certain parts of interesting BRDF data for relatively small α, where α is defined as the angle between the beam and the normal to the surface in the point of incidence. This problem can be explained by the device principle of operation (Figure 1a).

The inner side of the integrating hemisphere is a diffusing coating for wavelengths generated by a source of light (SOL). In its central part there is an aperture (AP) where the front of the sample under test (SUT) is mounted. The light beam in the angular range α from 0° to 90° is directed at the sample and reflects from it. By using a convex mirror (M) with an appropriate radius of curvature (following the fish-eye principle), a CCD camera (C) images the entire interior of the hemisphere, except for the area where the SOL is currently located. That area appears as a dark rectangle in the BRDF plot (Figure 1a).

## 3. Data Processing Algorithm

### 3.1. Raw Measurement Data

**Figure 2:**
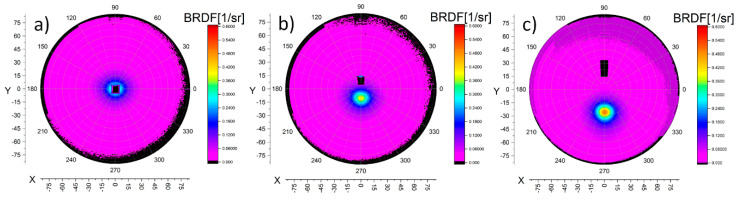
BRDF plot of the S420MC_oxidized sample for different α: (**a**) 0°, (**b**) 10°, and (**c**) 25°.

A result of the scatterometry measurement is a set of points defined in spherical coordinates (θ, ϕ, R) extended by the BRDF value; θ represents the azimuth angle, ϕ is the inclination angle and R is the radius of the integrating hemisphere. As R is constant for all of the points, results can be projected on the (θ, ϕ) plane for improved readability; as a consequence, the BRDF plots as a function of α are obtained (Figure 2). Overall, the BRDF function is defined as a ratio between the radiance reflected from a finite area S of the sample in one direction (θ*_r_*, ϕ*_r_*) to the incident irradiation from direction (θ*_i_*, ϕ*_i_*) [21]. The BRDF unit is steradian^−1^. In the case of IS-SA, θ*_i_* is constant and equals 0° whereas ϕ*_i_* is the same as α ranging from 0° to 90°. Moreover, the S area is defined by the AP (Figure 1a), which may have different diameters, depending on the user’s choice. It is worth underlining that while both the inclination angle and illumination angle are measured around the same axis, they are not the same angle. The first angle refers to the pixel position on the CCD camera, while the second one describes the position of the SOL in reference to the normal axis of the material.

In case of α larger than 15°, the dark rectangle in the BRDF plot is not a problem due to the directivity of reflection distribution (Figure 2c). The smaller the α, the higher the impact of obscuring the BRDF plot by a hole constituting the location of the light source (Figure 2b). Finally, for the case where α = 0°, the negative obscuring effect has a significant impact (Figure 2a).

**Figure 3:**
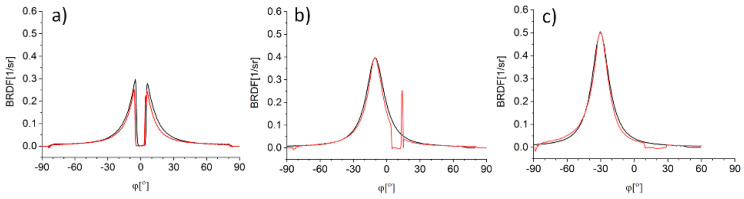
BRDF profiles of an S420MC_oxidized sample for different α: (**a**) 0°, (**b**) 10° and (**c**) 25°.

A cross section of BRDF data along the azimuth angle direction results in single BRDF profile which is a function of the inclination angle. Such profiles for three different α are presented in Figure 3 with respective cross sections of BRDF along the horizontal axis. Those horizontal cross sections are made at same inclination angle as α in order to include the maximum value of BRDF. Overlaying of the two profiles presented in Figure 3 supports the assumption that BRDF values are axis- symmetrical around the maximal value.

**Figure 4:**
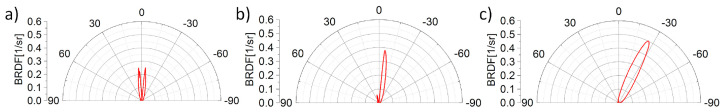
Reflection distribution (θ = 90°) of an S420MC_oxidized sample for different α: (**a**) 0°, (**b**) 10°, and (**c**) 25°.

The single BRDF profile along θ, given in polar coordinates (ϕ, BRDF), constitutes a cross section of reflection distribution, including comprehensive information about the directivity and intensity of the light reflected from the sample (Figure 4). This curve is similar to the light distribution curve which is often used in the literature to describe the emission of radiation from a light source [22].

The presented figures (Figure 2, Figure 3 and Figure 4) show that the results obtained for small α cannot be used for any analysis. One of the reconstruction methods of the BRDF profile based on the Pearson function fitted to incomplete data obtained for α = 0° was proposed by Reiner [23]. However, this method is not effective for samples characterized by narrow reflection distribution. Therefore, in the next section we propose a more universal approach for reconstruction of the BRDF data.

### 3.2. Algorithm Description

The scatterometry measurement results obtained for analyzed samples show that the BRDF is a combination of specular and diffuse reflection. The ratio between them varies, but for all samples the diffuse reflection was symmetrical in relation to direction (θ = 90°, ϕ = α) (Figure 3). Therefore, the assumption was made that the reflection distribution is symmetrical about the illumination axis (BRDF(α) = BRDF(-α)). Hence, each BRDF profile can be approximated with a single variable function. Parameters of these function could vary for different α but the function itself would not change. Due to the symmetry of reflection distribution about illumination axis the function parameters should also be symmetrical and reach extremal values at α = 0°. This allows interpolating or extrapolating parameters for the missing data.

In the first step of the algorithm, the BRDF profiles are extracted along the direction θ = 90°, but only for those α (≥15° in most cases) which the obscuring effect of the interesting BRDF data does not occur. Subsequently, the Lorentzian function (Equation (1)) is fitted to each of the BRDF profiles.

(1) $$BRDF\left(\Phi \right)=\left(BRDF_{MAX}-off\right)\frac{FWHM^{2}}{{\left(\Phi -\alpha \right)}^{2}+FWHM^{2}}+off$$

where *BRDF_MAX_* is the maximal value of *BRDF*, *FWHM* is the full width at half maximum, *ϕ* is the inclination angle, α is the illumination angle, and off is the offset.

**Figure 5:**
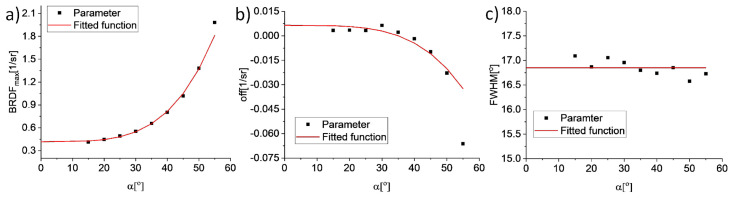
The fitted function of Lorentzian parameters versus α for the S420MC_oxidized sample. Parameters: (**a**) *BRDF_MAX_*, (**b**) off, and (**c**) full width at half maximum (*FWHM*).

In the algorithm, all values lower than 1% of *BRDF_MAX_* are labeled as background and ignored. The parameters of the fitted Lorentzian function are determined using the Levenberg–Marquardt method, which is a nonlinear optimization method that combines the steepest descent and Gauss–Newton methods. It was used to minimize the mean square error of fitting. Next, the relationship between the obtained Lorentzian parameters and α is fitted (Figure 5). The fitting curves for *BRDF_MAX_* and off points should be symmetrical in relation to ordinate axis. This condition forces the use of parity polynomials. Moreover, the 2nd order polynomial did not converge due to the high dynamics of the transition from the small changes in parameter values (<30°) to the area of rapid changes. Therefore, BRDF_MAX_ and off values are modelled with a 4th order polynomial. The *FWHM* values are fitted with a constant function due to its low variation.

**Figure 6:**
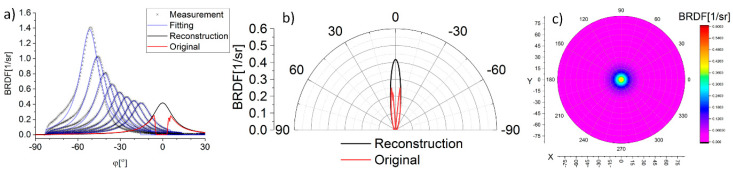
Original scatterometry measurement results with fitting for each α and reconstructed BRDF profile with comparison to original (**a**), reconstructed data for α = 0°: reflection distribution (θ = 90°) (**b**), and BRDF plot (compare with Figure 3a) (**c**).

Figure 6a shows the BRDF profiles along the direction θ = 90° that were obtained as a result of scatterometry measurements carried out for different measurements of α (gray cross markers). The result for α = 0° with the effect of obscuring is plotted in red. For all illumination angles (except for α = 0°) Lorentzian functions are fitted to the BRDF profiles based on Equation (1) (blue curves). Subsequently, the values of the function parameters for α = 0° are determined based on an extrapolation approach. Finally, the BRDF profile is reconstructed (black curve) and presented in polar coordinates on Figure 6b. The reflection distribution is symmetric with respect to the α axis as it was explained above. Therefore, the reconstruction of the BRDF plot is performed by rotating a BRDF profile along the ϕ = 0° axis (Figure 6c).

### 3.3. Validation

**Figure 7:**
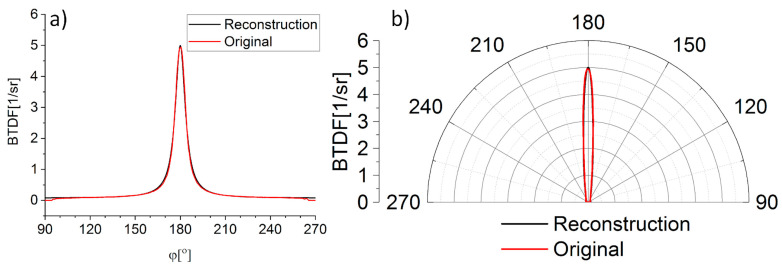
The algorithm validation by comparison of scatterometry measurement result and reconstruction for α = 180°: (**a**) bidirectional transmittance distribution function (BTDF) profile (θ = 90°), and (**b**) transmission distribution (θ = 90°).

In order to validate the developed reconstruction algorithm, transmission scatterometry measurements (BTDF) were carried out. The utilized sample was characterized by a 45% transmissivity level and a symmetrical transmission distribution. In the case of BTDF measurements, a light source is introduced from the outside of the integrating hemisphere (α > 90°). Therefore, the negative effect of obscuring the hemisphere by the light source does not occur. The measurements were carried out for α in a range from 120° to 180° with a 5° step. In the *BTDF* measurements the angle 180° corresponds to 0° in the BRDF measurements, with transmission instead of reflection. The developed algorithm was used to reconstruct the BTDF profile for α = 180° based on measurements for α from 120 to 165° with a 5° step. The BTDF profile measured for α = 180° and the one obtained as a result of the reconstruction are convergent in terms of amplitude and FWHM (Figure 7).

## 4. Results and Discussion

**Figure 8:**
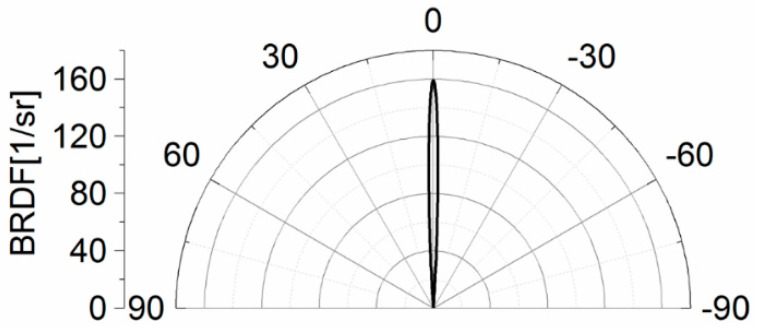
Reconstruction of the reflection distribution for the reference sample.

Firstly, the scatterometry measurements of the silver mirror as a reference sample were carried out. Subsequently, the reflection distribution for α = 0° was reconstructed based on the developed algorithm (Figure 8). The result of reconstruction confirms that the silver mirror is characterized by a narrow reflection distribution and high specular reflection (*BRDF_MAX_* = 160 steradian^−1^) [6].

**Figure 9:**
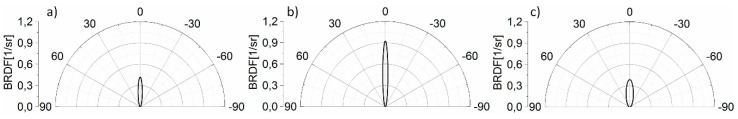
Reconstruction of the reflection distribution for samples: (**a**) S420MC_oxidized, (**b**) S420MC, and (**c**) S420MC_316L.

Subsequently, the scatterometry measurements and reconstruction process were carried out for a S420MC substrate sample with the surface preparation consisting only of degreasing (Figure 9a); in this case *BRDF_MAX_* = 0.42 steradian^−1^. The S420MC substrate after mechanical removal of the oxide layer is characterized by a much higher specular reflection (*BRDF_MAX_* = 0.92 steradian^−1^) (Figure 9b). Finally, in the process of laser metal deposition the functional coating (316L steel) was deposited on the S420MC substrate. The reconstructed reflection distribution obtained for the deposited coating is characterized by a much wider reflection distribution and specular reflection (*BRDF_MAX_* = 0.38 steradian^−1^) similar to the S420MC substrate (Figure 9c). The values of specular reflection obtained for all analyzed materials are considerably low in comparison with the silver mirror.

The reconstructed reflection distribution for α = 0° provides information about the total reflection intensity (*I_TOT_*) contained in a certain angle which depends on the surface of the material. The *I_TOT_* value obtained for the reference silver mirror was considered as I_REF_ due to a well-defined total reflectance higher than 97.5% for 575 nm. The results achieved for all analyzed materials, which were normalized to *I_REF_*, are presented in Table 2.

The mechanical removal of the oxidation layer from the surface of the sample S420MC increases the value of the parameter *I_TOT_*. Therefore, it can be concluded that the oxidized sample is characterized by a higher absorption than the same sample after removal of the oxidation layer. The highest value of *I_TOT_* was achieved for the laser deposited functional coating (S420MC_316L). However, for this material the widest reflection distribution was obtained (Figure 9c).

## 5. Conclusions

The purpose of the presented study was to determine the total reflection intensity from different materials commonly used in laser material processing. The described approach consisted of imaging scatterometry that was used for determination of the surface reflection distribution based on the obtained BRDF data. However, the BRDF data for a 0° illumination angle were useless. Therefore, an algorithm was developed to extrapolate the missing data of the reflection distribution for this problematic angle. A validation method based on the BTDF data was also proposed. The obtained validation results clearly indicate the reliability and effectiveness of the algorithm.

The scatterometry measurements were carried out for four materials in various surface conditions, i.e. silver mirror as a reference, two substrates made of mild steel S420MC and laser deposited functional coating made of stainless steel 316L.

The obtained results of total reflection intensity prove that the surface quality of the sample has the greatest impact on the considered intensity. The highest value of this intensity, as well as the widest reflection distribution was obtained for the laser deposited functional coating.

Further research will focus on extending the universality of the BRDF reconstruction algorithm to apply it to materials characterized by non-symmetrical diffuse reflection.

## Figures and Tables

**Table 1:** Surface parameters of analyzed samples.

Parameter	Designation (µm)	Silver Mirror	S420MC_oxidized	S420MC	S420MC_316L
Area root mean square height	Sq	0.04	2.66	8.32	12.05
Maximum peak height	Sp	3.49	30.40	47.80	112.45
Maximum pit height	Sv	2.18	21.50	63.90	87.63
Maximum height	Sz	5.67	52.00	112.00	200.09
Arithmetical mean height	Sa	0.02	2.06	6.35	8.26

**Table 2:** The total reflection intensity obtained for the analyzed samples.

Material	*I_TOT_* (%)
Silver Mirror (*reference*)	100.0
S420MC_oxidized (*substrate*)	12.1
S420MC (*substrate*)	18.4
S420MC_316L (*coating*)	38.3
